# use disorder in patients with autoimmune diseases:sociodemographic profile

**DOI:** 10.1192/j.eurpsy.2023.1611

**Published:** 2023-07-19

**Authors:** S. Stati

**Affiliations:** psychiatrie, faculté de medecine et de pharmacie Rabat, salé, Morocco

## Abstract

**Introduction:**

Autoimmune diseases are chronic and disabling conditions, especially because of the chronic pain they cause. Substance use disorders are on the rise in these patients, especially the problematic use of prescribed and over-the-counter analgesics.

**Objectives:**

to study the socio-demographic profile of patients with comorbidity between substance use disorders and autoimmune diseases, to assess the reasons for admission, the length of hospitalization, and the main psychoactive substances found in these patients

**Methods:**

A retrospective cross-sectional study of the medical records of patients with substance use disorders co-morbid with autoimmune diseases who were hospitalized in the addictology department of the ar-Razi University Psychiatric Hospital in Salé between January 2014 and December 2021.

**Results:**

10 patients were included in our study, the median age was 42, 76% were male, 41.3% were single, 63% had an average socio-economic level. 43.5% of the patients had a medical history, the main reason for admission was depressive syndrome (50%), the most common autoimmune disease was insulin-dependent diabetes, followed by IBD

**Conclusions:**

the comorbidity of autoimmune diseases and substance use disorders suggests the existence of common etiopathogenic mechanisms, the management of this comorbidity requires multidisciplinary collaboration

**Disclosure of Interest:**

None Declared

